# Retrospective analysis of skin complications related to bone-anchored hearing aid implant: association with surgical technique, quality of life, and audiological benefit^☆^

**DOI:** 10.1016/j.bjorl.2017.03.012

**Published:** 2017-04-25

**Authors:** Daniel Peñaranda, Juan Manuel Garcia, Maria Leonor Aparicio, Felipe Montes, Clemencia Barón, Roberto C. Jiménez, Augusto Peñaranda

**Affiliations:** a Universidad de los Andes, Facultad de Medicina, Bogotá, Colombia; b Fundación Santa Fe de Bogotá, Grupo de Implante Coclear, Bogotá, Colombia; c Fundación Universitaria de Ciencias de la Salud, División de Otología y Neurotología, Bogotá, Colombia; d Universidad de los Andes, Departamento de Ingeniería Industrial, Bogotá, Colombia

**Keywords:** Skin complications, Quality of life, Bone-anchored hearing aid, BAHA, Surgical technique, Complicações cutâneas, Qualidade de vida, Prótese auditiva óssea, BAHA, Técnica cirúrgica

## Abstract

**Introduction:**

The bone-anchored hearing aid is an effective form of auditory rehabilitation. Due to the nature of the implant, the most common complications are skin related. A number of alternative surgical implantation techniques have been used to reduce the frequency and severity of skin complications, including the U-shaped graft and the linear incision.

**Objective:**

To assess skin complications and their association with surgical technique, quality of life, and audiological benefit in patients with bone-anchored hearing aids.

**Methods:**

This was a retrospective study conducted in a tertiary referral center in Bogotá, Colombia. Patients who had been fitted with a bone-anchored hearing aid implant (unilaterally or bilaterally) for at least 6 months were included in the study. The Holgers classification was used to classify skin complications (Grade 0 = none; Grade 1 = erythema; Grade 2 = erythema and discharge; Grade 3 = granulation tissue; and Grade 4 = inflammation/infection resulting in the removal of the abutment). The Glasgow Benefit Inventory questionnaire was used to determine quality of life, and the Abbreviated Profile of Hearing Aid Benefit questionnaire was used to determine the subjective audiological benefit.

**Results:**

A total of 37 patients were included in the study (30 with unilateral implants and 7 with bilateral implant). Of the 44 implants evaluated, 31 (70.3%) were associated with skin complications (7 [15.9%] Grade 1; 4 [9.1%] Grade 2; 15 [34.1%] Grade 3, 5 [11.4%] Grade 4). The U-shaped graft was statistically associated with major complications (Grades 3 and 4) compared with the linear incision technique (*p* = 0.045). No statistically significant differences were found between Abbreviated Profile of Hearing Aid Benefit scores and severity of complications. Similarly, no differences were found between Glasgow Benefit Inventory physical health questions and skin complications.

**Conclusion:**

Despite the high frequency, skin complications did not seem to affect quality of life or subjective audiological benefits of patients with bone-anchored hearing aids.

## Resumo

**Introdução:**

A prótese auditiva óssea (BAHA, do inglês *Bone-Anchored Hearing Aid*) é uma forma eficaz de reabilitação auditiva. Devido à natureza do implante, as complicações mais comuns são relacionadas à pele. Várias técnicas alternativas de implantação cirúrgica têm sido utilizadas para reduzir a frequência e a gravidade das complicações cutâneas, incluindo o enxerto em forma de U e a incisão linear.

**Objetivo:**

Avaliar as complicações cutâneas e sua associação com a técnica cirúrgica, qualidade de vida e benefício audiológico em pacientes com BAHAs.

**Método:**

Esse é um estudo retrospectivo realizado em um centro terciário de referência em Bogotá, Colômbia. Os pacientes que receberam um implante de BAHA (unilateral ou bilateralmente) durante pelo menos 6 meses foram incluídos no estudo. A classificação de Holgers foi utilizada para classificar as complicações cutâneas (Grau 0 = nenhuma, Grau 1 = eritema, Grau 2 = eritema e secreção, Grau 3 = tecido de granulação e Grau 4 = inflamação/infecção resultando na remoção da estrutura de apoio). O questionário *Glasgow Benefit Inventory* (GBI) foi utilizado para determinar a qualidade de vida, e o questionário *Abbreviated Profile of Hearing Aid Benefit* (APHAB) foi utilizado para determinar o benefício audiológico subjetivo.

**Resultados:**

Um total de 37 pacientes foi incluído no estudo (30 com implantes unilaterais e 7 com implantes bilaterais). Dos 44 implantes avaliados, 31 (70,3%) foram associados às complicações cutâneas (7 [15,9%] Grau 1; 4 [9,1%] Grau 2; 15 [34,1%] Grau 3, e 5 [11,4%] Grau 4). O enxerto em forma de U foi estatisticamente associado a complicações maiores (Graus 3 e 4) em comparação com a técnica de incisão linear (*p* = 0,045). Não foram encontradas diferenças estatisticamente significativas entre os escores APHAB e gravidade das complicações. Do mesmo modo, não foram encontradas diferenças entre as questões de saúde física pelo questionário GBI e complicações cutâneas.

**Conclusão:**

Apesar da alta frequência, as complicações cutâneas não parecem afetar a qualidade de vida ou os benefícios audiológicos subjetivos de pacientes com BAHAs.

## Introduction

The bone-anchored hearing aid (BAHA) has proved to be effective in auditory rehabilitation. The device was introduced by Tjellström and Carlsson in 1977,^1^ and was initially approved for conductive and mixed hearing loss. More recently, the implants have been accepted for bilateral and sensorineural hearing loss.^2^, ^3^, ^4^ Several studies have reported improvements in quality of life as well as subjective and objective audiological benefits in patients fitted with the implant(s).^4^, ^5^, ^6^ Even patients with congenital abnormalities, such as aural atresia, are reported to benefit from BAHA implants.^7^

The BAHA is an acoustic amplification system consisting of three elements: a titanium fixture implanted in the mastoid process of the temporal bone, a skin abutment, and a sound processor anchored to the abutment. Due to the inherent contact of the implant with the skin, the most common complications are skin related, such as erythema, inflammation and infection. Severe complications may require the removal of the skin abutment.^8^

Several surgical modifications to the implantation technique have been used in an attempt to reduce the number of skin complications. These include the U-shaped graft and the linear incision techniques.^9^, ^10^ Our center, a tertiary referral center, has specifically used these two surgical techniques in previous years, and we have observed differences between the two techniques in terms of number and severity of skin complications.

To date, no statistical associations have been made between the type of skin complications and the quality of life or the subjective audiological benefits in patients with BAHA implants using validated questionnaires. A recent meta-analysis of complications associated with osseointegrated hearing aids suggested that lack of acoustic benefit and social considerations play an important role in deciding to become a non-user.^8^ Thus, it is possible that patients who have experienced a higher degree of skin complications may indicate a worse subjective audiological benefit or a lower quality of life.

The aims of the current study were to evaluate the frequency and severity of skin complications following BAHA implantation, and to investigate possible associations between skin complications and the surgical technique used, patient quality of life, and subjective audiological benefit.

## Methods

### Study design and patients

This was a retrospective study conducted in an otologic referral center (Otolaryngology Department, Hospital Universitario Fundacion Santafe, Universidad de Los Andes, Bogotá, Colombia). The study included patients who had been fitted with a BAHA device (either unilaterally or bilaterally) between 2003 and 2011. In order to minimize “enthusiasm bias”, only patients who had had the device for at least six months were included. There were no exclusion criteria for participation in this study. All of the patients were operated on by two otologists (A.P. or J.M.G) of the Otology Center in the University Hospital Fundacion Santafe, Bogotá, Colombia. The study was approved by the ethics committee of Fundacion Santafe de Bogotá. Patient consent was not needed due to the retrospective nature of the study.

Two different surgical techniques were employed in the study. The U-shaped graft technique with BAHA dermatome^9^ was used in 20 patients, 6 of whom received bilateral implants. A skin transplant was harvested and complete soft tissue removal to the periosteum was performed under the skin transplant area. After the drilling and insertion of the implant were performed, a hole was punched for the abutment and the split skin transplant was sutured tightly to the periosteum.^11^

The linear incision technique^10^ was used in 15 patients, all of whom received unilateral implants. In this technique, an incision approximately 2.5 cm in length was made and soft tissue was mobilized and raised over the periosteum at the implant site, but all hair follicles were left intact. A hole was punched for the abutment at 0.5–1 cm from the incisional line, leaving an intact periosteum except at the site where the fixture is inserted. The incision was sutured only at skin level, with no sutures to the periosteum.^11^

All patients were fitted with the 5 mm abutment. The tioblast implant surface was not used in any of the implants. Perioperative and postoperative antiseptics were not used. The bandage consisted of gauze dressing between the skin and a healing cap impregnated with an antiseptic ointment (Bactigras, Smith and Nephew, Canada), which were kept in place for 48 h. Patients were fitted with the processor between the third and fourth week after the surgery.

Information on the surgical technique for two of the patients (one patient with bilateral BAHA) was not available as they underwent surgery at different hospitals. Therefore, we analyzed 26 implants with the dermatome technique, 15 implants with linear incision technique, and 3 implants were the technique information was unavailable, for a total of 44 implants in 37 patients.

#### Evaluation of skin complications

The Holgers classification was used to evaluate skin complications^12^: Grade 0 = no adverse reaction; Grade 1 = skin with erythema; Grade 2 = skin with erythema and discharge; Grade 3 = granulation tissue; and Grade 4 = inflammation/infection resulting in the removal of the abutment. Skin complications were divided into minor (Grades 1 and 2) and major (Grades 3 and 4) complications based on the severity and the need for revision surgery.

All patients operated between 2003 and 2011, were invited to a follow-up consultation during which a photographic record of the implant was made in order to objectively assess the grade of the complication. Subsequently, patients were asked to indicate which skin complications they had ever experienced by using photographs of complication grades according to the Holgers classification. We revised the medical history notes of each patient in order to ensure that all complications were recorded. These medical histories also provided a record of patient adherence to follow-up visits and treatment.

A patient questionnaire, developed by the authors of this study, was used to determine subjective advantages and disadvantages of the device, as well as the need for treatment for the different skin complications (refer to Column 1) (Table 2).

#### Evaluation of quality of life

Quality of life after surgery was determined using the Glasgow Benefit Inventory Questionnaire (GBI).^13^ The GBI is a retrospective generic quality-of-life questionnaire developed by Robinson et al. to measure outcomes after otorhinolaryngologic procedures. This study has been previously validated.^5^ It is sensitive to changes in health status that result from an intervention, and it enables comparisons between different interventions.

In this questionnaire, 18 items cover three domains; 12 items are related to general improvement, 3 to social improvement, and 3 to physical improvement. Responses are given using a 5 point Likert scale. The total score calculations vary from −100 (maximum lack of benefit) to +100 (maximum benefit), with a score of 0 meaning no benefit. For the current study, we used the Spanish translation provided by the Institute of Hearing research website.^14^

Only the questions that focused on the health status of the patients and the use of treatment or medications were analyzed in the current study. The questionnaire was self-administered, but supervised by an audiologist and medical student during a scheduled post-surgery consultation to provide any clarification if required. We decided to use these specific questions for analysis in order to examine whether higher grades of complications were associated specifically with the use of treatment or attendance at follow-up consultations.

#### Evaluation of subjective audiological benefit

To investigate whether subjective audiological benefits were associated with skin complications, we used the Abbreviated Profile of Hearing Aid Benefit (APHAB),^6^, ^15^ which is a hearing disability questionnaire consisting of 24 questions covering four subscales. The APHAB outcomes are scored for unaided and aided conditions, and benefit is calculated by comparing the patient's reported difficulty in the unaided condition with their difficulty with amplification.

Three of the subscales address speech understanding in various everyday environments: ease of communication (EC, under relatively favorable conditions), listening under reverberant conditions (RV, communication in reverberant rooms), and listening in background noise (BN, in settings with high background noise levels). The Aversiveness (AV) of sounds subscale measures the negative reactions to environmental sounds.

The APHAB has a scoring scale from 1 to 99; the higher the score, the greater the hearing disability. An overall difference in the unaided and aided scores of more than 10 points for a given subscale (EC, RV, BN, and AV) was considered to be statistically significant.^12^ For the current study, we used the Spanish version of the APHAB provided by the University of Memphis website.^16^

### Statistical analysis

Individual patient data were coded and analyzed anonymously. The skin complications were explored using the Holgers classification system using a descriptive approach. The patient questionnaire results were analyzed using frequency tables.

Documented parameters in the patient questionnaire included: daily use of BAHA system, self-report of skin complications, attendance at medical consultations for skin complications, treatment and improvement of complications, replacement of abutment and processor, and self-perceived benefits and disadvantages of the BAHA system.

Comparisons of the skin complications by type of surgical technique were assessed using Fisher's exact test. Non-parametric analysis, using the Mann–Whitney *U* and Wilcoxon tests, were used for the comparison of APHAB and GBI results with skin complications. A level of significance of *α* = 0.05 was implemented, and the software used was Stata 10.0 and Mathematica 9.

## Results

A total of 37 patients (44 implants) were included in the study. The male:female ratio was 21:16, and the patients’ ages at initiation of the study ranged from 9 to 63 years.

Prior to surgery, 18 patients (48.6%) experienced mixed hearing loss in the implanted ear, 15 (40.5%) presented with conductive hearing loss, and 4 (10.8%) presented with sensorineural hearing loss.

In patients with a unilateral implant (*n* = 30), the hearing level in the contralateral ear was mixed hearing loss in 14 (48.3%), normal hearing in 6 (20.7%), sensorineural hearing loss in 5 (17.2%), and conductive hearing loss in 4 (13.8%).

The etiologies of the hearing loss in the implanted ear are summarized in Table 1. Of note, one patient had both chronic otitis media and a mastoidectomy, and 21 patients had external auditory canal agenesia.

**Table 1:** Baseline characteristics of patients.

Variable	Category	*n* (%)
Age of activation	Median (range)	32 (9–63)
Gender	Male	21 (57%)
	Female	16 (43%)
Amplification	Unilateral	30 (81%)
	Bilateral	7 (19%)
Etiology	Bilateral microtia	11 (29%)
	Unilateral microtia	10 (26%)
	Bilateral mastoidectomy	5 (13%)
	Sensorineural hearing loss	4 (10%)
	Chronic otitis media	2 (5%)
	Teacher Collins syndrome	2 (5%)
	Pfiffer syndrome	2 (5%)
	Unilateral mastoidectomy	2 (5%)
Hearing loss in the ear with the device	Mixed	18 (49%)
	Conductive	15 (41%)
	Sensorineural	4 (10%)
Unilateral patients-hearing in contralateral ear	Mixed	14 (48%)
	Normal	6 (21%)
	Sensorineural	4 (17%)
	Conductive	4 (14%)
Processor	Bp100	22 (61%)
	Divino	12 (33%)
	Ponto	2 (6%)

### Skin complications

#### Results from the patient questionnaire

Results from the patient questionnaire showed that 33 patients (89.2%) reported using the BAHA for more than 4 h a day, 3 patients (8.1%) used the device between 2 and 3 h a day, and 1 patient (2.7%) reported using the device between 1 and 2 h a day (Table 2). The questionnaire results showed that 28 patients (75.7%) used the BAHA system 7 days a week, 2 patients (5.4%) used it 6 days a week, and 7 patients (18.9%) used the device between 2 and 5 days a week. A total of 29 patients (78.4%) subjectively reported experiencing a skin complication; all of these patients received treatment with topical steroid creams, which improved the skin symptoms. Feedback noise and esthetics, which were reported in 40.4% and 14.8% of patients, respectively, were considered to be the most unpleasant factors associated with the BAHA.

**Table 2:** Patient questionnaire.

Question	Response	*n* (%)
How many hours a day do you use the BAHA?	1–2 h a day	1 (2.7%)
	2–3 h a day	3 (8.1%)
	>4 h a day	33 (89.2%)
How many days a week do you use the BAHA?	2–5 days a week	7 (18.9%)
	6 days a week	2 (5.4%)
	7 days a week	28 (75.7%)
Have you experienced any skin complications?	Yes	29 (78.4%)
	No	8 (21.6%)
		
If yes, have you gone to medical consultation for this skin complication?	Yes	29 (100%)
	No	0
		
Did treatment improve the skin complication?	Yes	28 (96.5%)
	No	1 (3.5%)
How many times have you changed your abutment?	Never	5 (13.5%)
	Once	32 (86.4%)
How many times have you changed your processor?^a^	Never	12 (33.3%)
	Once	12 (33.3%)
	Twice	7 (19.4%)
	Three times	1 (2.8%)
	Four times	2 (5.5%)
	Five times	1 (2.8%)
	Six times	1 (2.8%)
What do you most dislike about the BAHA?^b^	Feedback noise	19 (40.4%)
	Esthetically unpleasant	7 (14.8%)
	Skin complications	4 (8.5%)
	Stability of the processor^c^	14 (29.8%)
	Nothing	3 (6.3%)

a Only 36 patients were taken into account in this question.

b Patients could select multiple answers in this question.

c Stability refers to easily falling off and/or the duration of batteries.

#### Severity of skin complications

**Figure 1:**
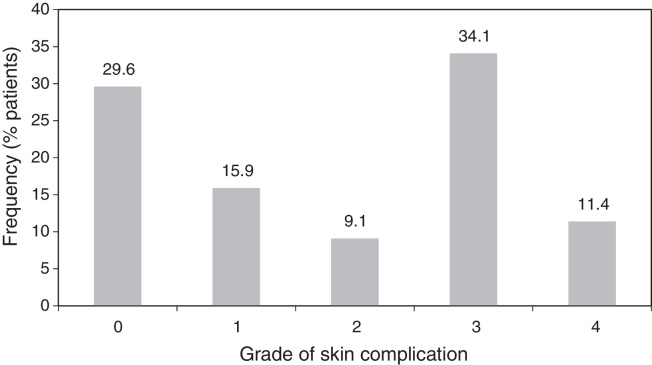
Severity of skin complications.

In our analysis of the 44 implants (37 patients), 13 implants (29.6%) did not experience any skin complication (Grade 0), 7 implants (15.9%) had experienced Grade 1 skin complications, 4 implants (9.1%) Grade 2, 15 implants (34.1%) Grade 3 and 5 implants (11.4%) Grade 4 skin complications (Fig. 1). Overall, 45.5% of the implants were associated with major skin complications and required revision surgery.

### Association of skin complications with surgical technique

**Figure 2:**
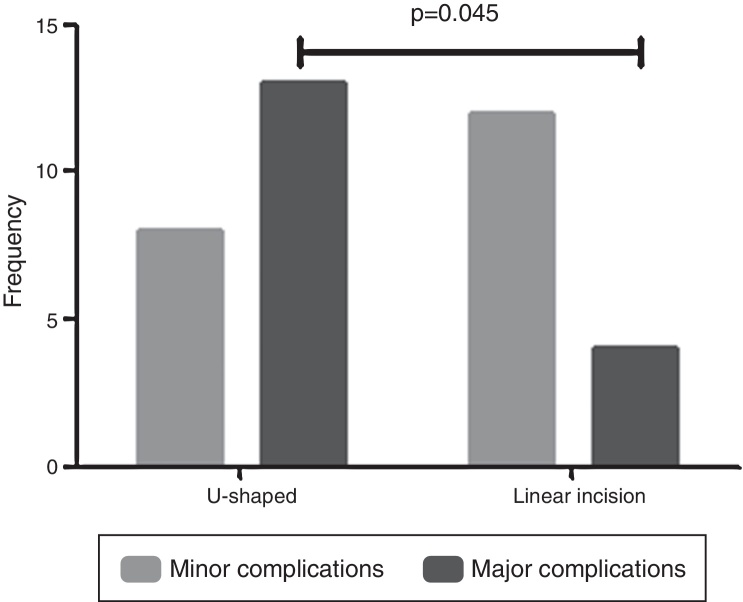
Frequency of minor and major complications by intervention technique.

Statistically significant differences in the severity of skin complications were found between the surgical techniques used for BAHA implantation, according to the Fisher's exact test (Fig. 2). In fact, the U-shaped graft technique with BAHA dermatome was statistically associated with major complications compared with the linear incision technique (*p* = 0.045).

### Association of skin complications with subjective audiological benefit

No statistically significant differences were found between APHAB global score or subscales and severity of complications The *p*-values of the Mann–Whitney *U* statistical difference test were >0.05 for the global score and each of the subscales (EV score *p* = 0.0769, BN score *p* = 0.646, RF score *p* = 0.087, AV score *p* = 0.190, Global score *p* = 0.270).

### Association of skin complications with quality of life

**Figure 3:**
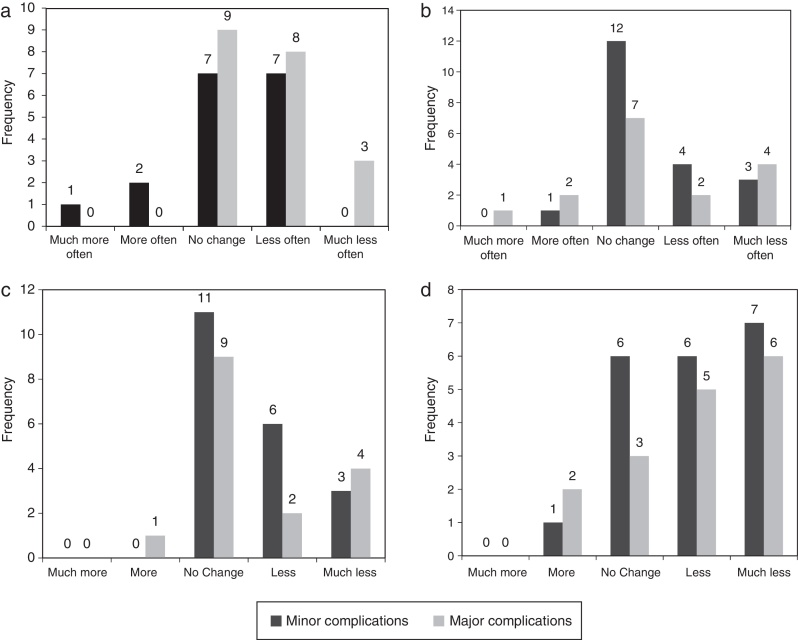
Frequency of each option answered by patients presenting minor and major skin complications for the Glasgow Benefit Inventory questions related to health status and the use of treatment or medications: (a) Question 8: Have you been to your family doctor, for any reason, more or less often, since your operation/intervention; (b) Question 12: Since you had the operation/intervention, do you catch colds or infections more or less often?; (c) Question 13: Have you had to take more or less medicine for any reason, since your operation/intervention?; (d) Question 16: Since your operation/intervention, are you more or less inconvenienced by your health problem?

No statistically significant differences were found between any of the four GBI physical health questions results (questions 8, 12, 13, and 16) and the severity of skin complications (Fig. 3). For each of the questions, the *p*-values of the Wilcoxon test were >0.05 (*p* = 0.113, 0.848, 0.806, and 0.988, respectively).

## Discussion

The aims of this study were to assess the severity of skin complications related to BAHA implants and to investigate whether there was an association between skin complications and type of surgery, subjective audiological benefit, and quality of life.

In terms of severity of skin complications, 45.5% of patients reported having Grade 3 or Grade 4 complications. This is a high rate of major complication compared with those quoted in the literature (9.4–84%).^17^ This difference may be explained by selection bias, as patients with more complications may be more likely to attend follow-up consultations. Almost all of the patients who experienced skin complications showed an improvement in skin symptoms with the use of medical treatment. It is worth mentioning that skin complications were not viewed as a major unpleasant factor of the BAHA system in the current study; feedback noise and the look of the device were considered to be the most unpleasant factors of the BAHA.

The linear incision technique was statistically significantly associated with fewer major complications, which correlates with other reports. Van de Berg et al.^18^ compared four surgical techniques (retroauricular skin graft, skin flap, dermatome, and vertical incision) in a total of 143 patients, and found that the linear incision technique was significantly associated with fewer major complications (*p* = 0.0021), and with a shorter time until use of BAHA (2 months) compared with the other techniques. Wilkinson et al.^10^ reported a complications rate of 16.9% in 71 patients for the vertical incision. De Wolf et al.^19^ reported skin reactions in a total of 1038 observations from 150 patients (16.6% of the observations), with the majority (10.1%) being Holgers Grade 1. Faber et al.^20^ observed skin reactions in 130 patients (52.4% of the total sample), and 18.6% had a major complication (Holgers Grades 2–4). In their recent systematic review, Mohamad et al.^21^ concluded that the use of linear incision appeared to be associated with fewer complications, but highlighted the need for uniform reporting standards.

Newer techniques have been introduced in order to reduce the frequency of complications, such as the BAHA implantation without tissue reduction introduced by Hultcrantz and Lanis.^22^ This technique has exhibited better outcomes in comparison with the dermatome technique^22^; however, outcome comparisons between the linear incision with and without tissue reduction remain to be elucidated.

Our study also sought to correlate skin complications with subjective audiological benefits and quality of life after surgery. Indeed, we hypothesized that a person with major complications and requiring revision surgery would have alterations in their quality of life and subjective audiological benefits, as reported by the GBI and APHAB questionnaires. However, we did not find statistically significant associations between GBI and APHAB results, and skin complications. Again, although BAHA skin complications are the most frequent complications, these do not seem to influence the patient's subjective audiological benefits or the quality of life after the surgery. Strengths of this study include the use of validated quality-of-life and auditory questionnaires and the evidence of the most positive and negative BAHA factors based on the patient's perspective. Limitations include the retrospective study design, the fact that it was a single center study and the subjective nature of the measures. In light of this, more studies with a larger number of patients are needed to clarify these associations.

## Conclusion

Despite the high frequency of skin complications associated with BAHAs in our cohort, patients appeared to be satisfied with the device and skin complications did not seem to affect quality of life or the subjective audiological benefits. Based on our cohort of patients, we recommend the use of the linear incision since it was associated with fewer skin complications in comparison with the U-dermatome. Further analysis will include larger samples with longer follow-ups, and comparisons with the new transcutaneous implants.

## Conflicts of interest

The authors declare no conflicts of interest.
